# Long-Acting Cabotegravir/Rilpivirine for HIV Antiretroviral Therapy Throughout Pregnancy: A Case Report and Literature Review

**DOI:** 10.1155/crdi/6207502

**Published:** 2025-11-12

**Authors:** Sarah Boudova, Karley Dutra, Carol Lynn Robbins, Joy Lander-Roe, Stephen Pagkalinawan, Rodney A. McLaren

**Affiliations:** ^1^Department of Obstetrics and Gynecology, Division of Maternal Fetal Medicine, Thomas Jefferson University, Philadelphia, Pennsylvania, USA; ^2^Department of Obstetrics and Gynecology, Thomas Jefferson University, Philadelphia, Pennsylvania, USA; ^3^Drexel University College of Medicine, Philadelphia, Pennsylvania, USA; ^4^Division of Infectious Diseases and HIV Medicine, Partnership Comprehensive Care Practice, Drexel University College of Medicine, Philadelphia, Pennsylvania, USA; ^5^Department of Obstetrics and Gynecology, Division of Maternal Fetal Medicine, Thomas Jefferson University, Philadelphia, Pennsylvania, USA

**Keywords:** antiretroviral therapy, cabotegravir, case report, HIV, perinatal transmission, pregnancy, rilpivirine

## Abstract

Cabotegravir/rilpivirine (CAB/RPV) is a long-acting injectable antiretroviral therapy (ART) with limited data on safety and efficacy during pregnancy. We report a patient who maintained viral suppression on CAB/RPV every 2 months throughout pregnancy. MV was a 33-year-old G4P1021 with HIV and hypertension. She was infected perinatally and treated with multiple ART regimens due to inconsistent adherence to oral ART. Prior to this pregnancy, she switched to long-acting intramuscular CAB (600 mg)/RPV (900 mg), which allowed her to maintain an undetectable viral load. In preconception counseling, we advised her of the unknown efficacy and safety during pregnancy. She elected to continue CAB/RPV every 2 months and maintained 100% adherence with an undetectable viral load throughout pregnancy. Fetal pyelectasis was identified by ultrasound and persisted throughout pregnancy. A male infant with growth restriction (2.33 kg) was delivered via scheduled 37-week cesarean and admitted to the neonatal ICU for respiratory support for two days. Postnatal ultrasound confirmed hydronephrosis. The infant had negative HIV testing at one, two, and 10 months. There are limited reports of CAB/RPV continuation throughout pregnancy. One previous report identified fetal growth restriction and ptosis of the neonate. Another reported normal birthweight and no congenital anomalies. Most recently, an abstract presented 22 pregnancies continued on CAB/RPV. Anomalies in this series were pyelectasis, ventriculomegaly, and Trisomy 21. None of these reports observed perinatal HIV transmission. Primary concerns for the use of CAB/RPV during pregnancy are the possibility of reduced drug efficacy and adverse neonatal outcomes. The existing literature is sparse, but multiple studies have shown reduced RPV concentrations during pregnancy, raising concern for ineffective viral suppression. There is no evidence of teratogenic effects. Benefits of CAB/RPV during pregnancy include improved drug adherence and discretion. Based on our experience and review of the literature, long-acting intramuscular CAB/RPV may be of value in treatment-experienced patients experiencing difficulties with pill adherence.

## 1. Introduction

Globally, 1.3 million pregnancies per year are estimated to occur among persons living with HIV, and 120,000 cases of perinatal infection occur [1, 2]. Antiretroviral therapy (ART) has significantly decreased the rate of perinatal transmission and is recommended for use throughout pregnancy by the Society for Maternal–Fetal Medicine, the National Institutes of Health, and the World Health Organization [1, 3, 4]. Perinatal transmission rates are less than 1% for individuals who remain on ART with an undetectable viral load (< 50 copies/mL) throughout pregnancy [5]. However, inconsistent adherence to ART remains a significant challenge for certain individuals, increasing the risk of perinatal HIV transmission [6]. Switching ART during pregnancy is another vulnerable time, as patients may develop viremia, increasing perinatal transmission.

Cabotegravir/rilpivirine (CAB/RPV) is a long-acting injectable ART administered monthly or every other month, which can lead to improved ART adherence [7, 8]. CAB is an integrase strand transfer inhibitor, and RPV is a non-nucleoside reverse transcriptase inhibitor. Monthly and every-2-month CAB/RPV injections have been shown to be noninferior to oral ART at suppressing HIV-1 in nonpregnant adults [9, 10]. There are limited data on the safety and efficacy of CAB/RPV during pregnancy. Studies have largely focused on individuals who became pregnant while on CAB/RPV and transitioned to oral ART for the remainder of pregnancy [11–13], with a handful of reports of individuals who continued CAB/RPV throughout pregnancy [13–15]. Here, we describe a patient who received long-acting CAB (600 mg)/RPV (900 mg) injections every 2 months throughout pregnancy, followed by a literature review on CAB/RPV use during pregnancy.

## 2. Case Presentation

MV was a 33-year-old G4P1021 with a pregnancy complicated by HIV, chronic hypertension, hemoglobin C trait, asthma, and a history of cesarean delivery. Her first pregnancy was a spontaneous abortion managed with a dilation and curettage, her second pregnancy was an ectopic pregnancy treated with methotrexate, and her third pregnancy was a 38-week uncomplicated cesarean delivery in 2016.

With regards to her HIV history, MV was perinatally infected and had previously received multiple ART regimens. Her CD4 nadir was 81 in 2022, and she never had any opportunistic infections. She received opportunistic infection prophylaxis when indicated. Her childhood records were not available; however, per chart review, at unspecified time periods, she was on combined efavirenz, emtricitabine, and tenofovir disoproxil fumarate; combined elvitegravir, cobicistat, emtricitabine, and tenofovir alafenamide; and darunavir. In 2014, she was on combined emtricitabine, tenofovir disoproxil fumarate, darunavir, and ritonavir. In 2015, she was on combined emtricitabine, rilpivirine, and tenofovir disoproxil fumarate. In 2016, she was started on combined elvitegravir, cobicistat, emtricitabine, and tenofovir disoproxil fumarate plus darunavir. In 2018, she had viral genotyping which showed resistance to tipranavir and ritonavir. In 2020–2022, she was on combined bictegravir, emtricitabine, and tenofovir alafenamide fumarate. With all of these regimens, she had a history of inconsistent adherence to oral ART. In March 2022, a plan was made to be started on long-acting injectable CAB/RPV. Her BMI was 27. Her CD4 count was 91, and her viral load was < 40 copies/m, with her last detectable viral load being 1630 copies/mL a month prior. The initial transition plan was for oral lead-in for 2 weeks followed by the first injection. However, her viral load at the time of the oral lead-in was 36,940 copies/mL, so the oral lead-in was extended to four weeks. Her repeat viral load at four weeks was 160 copies/mL. The viral genotype was ordered at this time but could not be run due to the lower viral load. She was initiated on injectable CAB/RPV, and her viral load was undetectable in June.

During her 2016 pregnancy, she was on elvitegravir/cobicistat/emtricitabine/tenofovir and darunavir as described above and struggled with adherence. She had a third-trimester viral load of 6141 copies/mL, leading to a 38-week cesarean delivery with intravenous zidovudine administration before delivery to reduce her risk of perinatal HIV transmission. The infant received postexposure prophylaxis and subsequently tested negative for HIV.

In 2023, while on CAB/RPV, MV desired to conceive again. She had preconception counseling by Maternal–Fetal Medicine and Infectious Diseases providers on the lack of safety and efficacy data for CAB/RPV use during pregnancy. Providers also reviewed that patients on CAB/RPV usually transition to oral ART before conception with a plan to return to CAB/RPV postpartum. MV was elected to continue CAB/RPV during pregnancy, deciding that the benefits of ART adherence outweighed the unknown risks of the medication. Respecting her autonomy, she was maintained on CAB/RPV.

After conception, she was followed by Maternal–Fetal Medicine, Infectious Diseases, Social Work, and Nursing in a comprehensive care program for pregnant individuals with HIV. Her antepartum course was complicated by fetal pyelectasis, chronic hypertension which was stable and well-controlled on amlodipine 5 mg once daily, and fetal growth restriction. She was diagnosed with moderate right-sided fetal pyelectasis at her 22-week anatomy ultrasound, with the renal pelvis measuring 8.9 mm. She met criteria for severe fetal pyelectasis at 33 weeks, with the renal pelvis measuring 22.4 mm and the kidney measuring 51.9 mm (> 95th percentile). The fetus was also diagnosed with fetal growth restriction (estimated fetal weight 7th percentile by Hadlock formula, with normal uterine artery Dopplers) at this time. The kidney and renal pelvis measurements remained stable throughout the remainder of her pregnancy. She maintained 100% adherence to CAB/RPV, receiving all doses on time, and her viral load was checked monthly and remained undetectable. Her CD4 count during pregnancy was 285 and 250 when checked. Her last antepartum viral load was checked 8 weeks prior to delivery. Viral load was repeated on admission for delivery and was undetectable. She had a scheduled repeat cesarean section in November 2023, at 37 weeks due to uncomplicated chronic hypertension controlled on medications. She delivered a liveborn male infant weighing 2.33 kg (7.8th percentile for gestation age) with Apgar scores of 5 and 8, at 1 and 5 min, respectively. The infant was admitted to the neonatal intensive care unit (NICU) for respiratory support requiring continuous positive airway pressure. He received 4 weeks of zidovudine prophylaxis (4 mg/kg every 12 h). He was weaned to room air and discharged from the NICU on Day 2 of life. MV had previously been counseled on the option for breastfeeding and declined. On postnatal ultrasound, the infant had a normal-sized right kidney with moderate to severe hydronephrosis. The infant has remained HIV negative by PCR testing at one, two, and 10 months.

## 3. Discussion/Conclusion

There are few reports of patients maintained on long-acting CAB/RPV throughout pregnancy [13–15]. Our patient's HIV viral load remained undetectable throughout pregnancy, and there was no perinatal transmission with negative infant testing at one, two, and 10 months of life, similar to previously published reports. In a case series of women in clinical trials exposed to CAB/RPV during pregnancy, one participant was continued on CAB/RPV with monthly dosing [13]. The pregnancy was complicated by fetal growth restriction, preterm birth, and ptosis (which was considered random by the treating physician and resolved on its own). The mother had a reduction in viral load but did not achieve an undetectable viral load. The infant received 4 weeks of triple therapy with zidovudine, lamivudine, and raltegravir. The infant tested negative for HIV at birth and 4 weeks of life. In a recent case report, a patient continued CAB/RPV dosing every 2 months during pregnancy. She maintained viral suppression from conception to delivery with no perinatal HIV transmission [14]. The infant had a normal birthweight and no congenital anomalies. The infant did not receive postpartum HIV prophylaxis because the mother had an undetectable viral load. HIV testing was negative at 1 week, and three and 6 months of life. Most recently, an abstract was presented with 22 cases of women continued on CAB/RPV during pregnancy [15]. Among these women, 90% maintained a viral load < 200 copies/mL. There were no cases of vertical transmission. Congenital anomalies included ventriculomegaly, pyelectasis, and Trisomy-21 which were not attributed to CAB/RPV.

One concern for antepartum continuation of CAB/RPV is altered efficacy of the drug due to the physiologic changes of pregnancy. Theoretically, reduced drug concentrations in pregnancy could result in less effective viral suppression, increased perinatal HIV transmission, and the development of drug resistance. In an abstract of physiologically based pharmacokinetic (PBPK) models with discontinuation of CAB/RPV during pregnancy, levels fell below established minimum effective concentration in most women after 33-week gestation [16]. In a pharmacokinetic modeling study of long-acting injectable CAB/RPV dosed every month and every other month using pregnancy-induced metabolic and physiologic changes, CAB C_trough_ levels were comparable to nonpregnant adults for both monthly and every-other-month dosing, indicating an ability to maintain efficacy, with the exception of reduced levels noted in the third trimester for individuals on every-other-month dosing [17]. In contrast, RPV C_trough_ dosing was below target in at least 40% of the pregnant population with monthly dosing, increasing to over 90% with every-other-month dosing. A small pharmacokinetic study of oral daily RPV showed decreased drug levels during pregnancy, but there were no safety concerns or cases of perinatal HIV transmission [18]. Another small study similarly demonstrated reduced RPV plasma concentrations after oral dosing [19]. Currently, there are no data to support increased oral dosing of RPV during pregnancy. An ex vivo placental perfusion model showed lower transfer of CAB than other ARTs, which could limit fetal toxicity but also limit the ability of the drug to prevent vertical transmission [20]. In the recent case study referenced above, CAB concentrations were comparable to those outside of pregnancy, although RPV levels were 70%–75% lower [14]. We did not examine drug levels in maternal or cord plasma in our patient. Although we maintained our patient on the every-2-month regimen, pharmacokinetic modeling suggests that once monthly injections may be more effective in pregnancy and that more frequent viral load monitoring should be considered, especially during the third trimester [17]. Further research is needed on the optimal frequency of dosing in pregnancy.

Another theoretical concern regarding antepartum CAB/RPV use is teratogenicity. Our patient's pregnancy was complicated by fetal pyelectasis and growth restriction. It is unclear if these findings were related to CAB/RPV exposure. Animal studies have not shown teratogenic effects of the CAB/RPV [13] nor have studies of early pregnancy exposure [11–13]. In the HPTN 084 randomized clinical trial evaluating CAB, 49 participants had a confirmed pregnancy and 29 were in the injectable CAB group [11]. No congenital anomalies were noted in the CAB group who had live births with outcomes available, and all individuals were taken off CAB and offered oral pre-exposure prophylaxis versus discontinuation. Similarly, follow-up from clinical trial data on individuals who became pregnant while on long-acting CAB/RPV is reassuring, with 25 pregnancies identified [13]. All but one individual switched to oral ART. The one infant with CAB/RPV exposure throughout pregnancy was noted to have congenital ptosis [13]. In the Antiretroviral Pregnancy Registry, congenital defects were reported in 3% of live births following *in utero* exposure to any ARTs during any trimester [21]. Our patient was enrolled in the Antiretroviral Pregnancy Registry (https://www.apregistry.com/). We recommend providers participate in the registry to collect additional information on pregnancies with exposure to CAB/RPV and other ART regimens. In the case reported by Patel et al. and for our patient, the pregnancy was complicated by fetal growth restriction [13]. For our patient, the impact of CAB/RPV exposure on fetal growth restriction is unclear given the confounding factors of chronic hypertension requiring medication and HIV on ART [22]. Her chronic hypertension was treated with amlodipine, which is not associated with fetal anomalies or growth restriction [23]. More data on the safety of long-acting CAB/RPV are needed, especially as even longer-acting formulations are in development [24].

Despite these potential concerns, long-acting CAB/RPV has several advantages. First, as our case illustrates, it can improve medication adherence through less frequent directly observed therapy rather than a daily pill. Second, an injection avoids issues with nausea, emesis, or difficulty swallowing pills, which are common problems in pregnancy [25]. Third, it provides discretion. If the patient's partner or family is unaware of her HIV status, confidentiality can be maintained by keeping ART medication out of the home.

Long-acting injectable CAB/RPV has the potential to overcome barriers to ART adherence in pregnancy. Our patient maintained 100% adherence and an undetectable viral load, without perinatal HIV transmission. However, further studies are needed on the safety and efficacy during pregnancy. Patients who elect to remain on CAB/RPV during pregnancy should be counseled on the limited data and managed with frequent viral load monitoring.

## Data Availability

Data are available on request due to privacy/ethical restrictions.
